# The Degree of Oxidation of Graphene Oxide

**DOI:** 10.3390/nano11030560

**Published:** 2021-02-24

**Authors:** Alexandra Carvalho, Mariana C. F. Costa, Valeria S. Marangoni, Pei Rou Ng, Thi Le Hang Nguyen, Antonio H. Castro Neto

**Affiliations:** 1 Centre for Advanced 2D Materials, National University of Singapore, Singapore 117542, Singapore; mariana.cfcosta@u.nus.edu (M.C.F.C.); valeriamarangoni@nus.edu.sg (V.S.M.); c2dnpr@nus.edu.sg (P.R.N.); hang@nus.edu.sg (T.L.H.N.); 2 Materials Science and Engineering, National University of Singapore, Singapore 117575, Singapore

**Keywords:** graphene oxide, composition, structure, XPS, theory, experiment

## Abstract

We show that the degree of oxidation of graphene oxide (GO) can be obtained by using a combination of state-of-the-art ab initio computational modeling and X-ray photoemission spectroscopy (XPS). We show that the shift of the XPS C1s peak relative to pristine graphene, $\Delta E_{C1s}$, can be described with high accuracy by $\Delta E_{C1s}=A{\left(c_{O}-c_{l}\right)}^{2}+E_{0}$, where $c_{0}$ is the oxygen concentration, A=52.3 eV, $c_{l}=0.122$, and $E_{0}=1.22$ eV. Our results demonstrate a precise determination of the oxygen content of GO samples.

Graphene oxide (GO) is an amorphous , non-stoichiometric, functionalized form of graphene bearing different oxygen functional groups [1,2,3]. Because GO disperses in water and other polar solvents, it has attracted enormous attention in scientific research and for technological applications [4,5]. However, the lack of control of GO stoichiometry, which is mostly determined by the oxygen atoms, leads to serious difficulties in the repeatability and reliability of experiments and industrial scaling of applications. Since some oxygen-related functional groups have similar signatures in infra-red absorption spectra or in X-ray photoelectron spectroscopy, it is at present difficult to quantify them independently. Furthermore, there are very few experimental techniques that can measure the degree of oxidation of GO with sufficient accuracy.

Here, we present a technique based on the analysis of the XPS peak energies that can quantitatively determine the oxygen content of GO. The main result is shown in Figure 1. The shift of the C1s XPS peak (relative to graphene) as a function of oxygen concentration (in theory and in the experiment) can be fitted by

(1) $$\Delta E_{C1s}=A{\left(c_{O}-c_{l}\right)}^{2}+E_{0}$$

where $c_{O}$ is the oxygen concentration, A=52.3 eV, $c_{l}=0.122$, and $E_{0}=1.22$ eV. Equation (1) determines the oxygen concentration by direct measurement of the C1s peak shift.

Graphene oxide dispersions with different degrees of oxidation were prepared by direct oxidation of graphene flakes using a modified Hummer’s method (see Methods section) [6]. The evolution of the oxygenated groups in the graphene structure with the degree of oxidation was evaluated experimentally by controlling the time of the oxidation reactions.

Figure 2 shows the XPS in each case. The spectra could be deconvoluted in 5 main peaks, which are usually attributed to 284.8 eV (C=C), 285.7 eV (C–C), 287.7 eV (C–O), 288.8 eV (C=O), and 289.8 (O–C=O) [7]. Comparing the spectra, we can clearly observe a relative increase in the oxygen-based groups. The percentage of C=C decreased from 81.8% in graphene to 9.4% in GO with the highest degree of oxidation, while the C–O peak increased from 2.8% to 46.6%. This also happened with C=O, which increased from 1.4% to 5.3%, and O–C=O, which changed from 0.7% to 4.4% (see Table S1). By increasing the oxidation, we also observed a relative blue shift and broadening, especially in the C–O peak. The C1s core level shift for C–O, for each one of these concentrations, with respect to the C=C shift, is shown as red circles in Figure 1. For comparison, we also show data for GO of two different commercial suppliers, from Reference [8]. As our fabricated GO is obtained from graphene, and not from graphite flakes, the oxidation process is faster and more homogeneous throughout its surface area. For this reason, this indicates better agreement with the theory.

The functional groups responsible for the C–O, C=O, and O–C=O peaks in XPS can be narrowed down to four candidates. These include the epoxy and hydroxyl groups at the basal plane (as in the model of Lerf et al. [9]), as well as a hypothetical carbonyl group resulting from the deprotonation of a hydroxyl. Such a carbonyl is, however, found to be unstable when isolated from other functional groups by DFT calculations (see Methods section). Figure 3a–c shows the structures of these three basal plane groups. Carboxyl groups, responsible for the O–C=O peak, form predominantly at the edges of the graphene oxide flakes or multivacancy clusters, such as the carboxyl groups shown in Figure 3d.

The C1s core level shifts for the four functional groups just discussed have been calculated using density functional theory, using a self-consistent ‘ΔSCF’ approach [10,11]. Previous work has shown that core level shifts can be obtained in the context of pseudopotential calculations, where only valence electrons are considered explicitly, yielding good agreement with all-electron calculations [12]. Our pseudopotential calculations also yielded excellent agreement with GPAW calculations [13].

Several approximations are involved in the ‘ΔSCF’ approach. We neglect any dynamics associated with the photo-emission process and assume that there is only one well-defined ground state. The binding energy ($E_{B}$) of the photo-emitted core electron is then the difference between the system with an ionized core ($E_{f}\left(A^{+}\right)$) and the ground-state energy of the system ($E^{0}$),

(2) $$E_{B}=E_{f}\left(A^{+}\right)-E^{0}.$$

This includes the effects of valence electron relaxation after the core ionization has taken place, but it does not include the relaxation of the lattice, which is expected to happen in a much slower time scale.

The ground-state energy of the system in the presence of the core hole is computed using a carbon pseudo-potential where the 1s level occupation has been decreased by one, and the valence occupation increased by one, obtained from the QuantumEspresso database, [14] with the type described in Reference [15]. Additionally, since graphene is semi-metallic, we assume that when a core electron is removed by photo-excitation, another electron is transferred from the detector, so that the system remains neutral. We adopt the same approximation for graphene oxide, since there is no evidence that the sample becomes charged after the experiments.

The C1s level shift of a C1s state hole near the oxygen functional group is compared to that of graphene. This eliminates the problem of lack of a common energy reference when different pseudopotentials are used. Further, it is directly comparable to the distance between the peak under consideration and the C=C peak for the same concentration.

Previous DFT works [13,16] have already thoroughly addressed the core level shifts of oxygen and hydrogen functional groups in graphene, the initial phase of oxidation of GO. One of the conclusions to note in References [13,16] is that, in graphene, the C1s core level shifts for a certain carbon atom do not depend only on the atoms that are directly bonded to it; atoms up to the fourth nearest neighbor of oxygen functional groups still display sizable C1s core level shifts. Further, the core level shift is not monotonic with the distance from the oxygen or hydrogen atom [17].

We have therefore calculated the C1s core level shifts up to the fourth nearest neighbor for basal plane functional groups, and up to the third nearest neighbor for the edge carboxyl (Figure 4a–d). In each case there were two peaks: a major peak nearer to 0 eV, corresponding to the less perturbed carbon atoms, and a smaller peak to the positive side corresponding to the first nearest neighbor (NN). The core level shifts were calculated using pristine graphene as reference (see Computational Methods). Our results were in excellent agreement with References [13,16] for epoxy, hydroxyl, carbonyl at the basal plane and carboxyl. We also calculated core level shifts for other carbonyl forms (not shown in Figure 4), including edge carbonyl, nearest-neighbor, and second nearest-neighbor carbonyl pairs, which were found to have C1s core level shifts of −0.89, 0.77, and 0.79 eV, respectively, relative to the graphene peak on the nearest carbon atom.

The results agreed well with the experimental spectra for graphene, namely for the C−O peak, which was the highest oxygen-related peak and was 1.48 eV higher in energy than the C-C $sp^{3}$ peak. This is very close to the value calculated for the epoxy (1.52 eV relative to the C-C $sp^{3}$ peak), whereas the calculated hydroxyl value was lower, 1.04 eV above the C-C $sp^{3}$ peak). We verify that, except for the edge carboxyl, C1s core level shifts of all these oxygen groups in graphene do not exceed 2 eV, whereas experimental shifts for carbonyl and epoxy are up to 3.9 eV and 3.4 eV, respectively.

The more oxidized the graphene is, the less metallic it becomes, and fewer free electrons are available to screen ionized cores. This induces the drift of the core level shifts that we report in this letter. We thus carried out calculations in a GO model consisting of a series of oxidized 32-atom graphene supercells, with randomly positioned, parallel-oriented epoxy oxygen, in a concentration of up to 33% atomic fraction (Figure 5). The use of the parallel epoxy is necessary to prevent the formation of pairs, thus isolating the effect of long-range interactions. For each oxygen concentration, the C1s core level shift was calculated for each of the 32 carbon atoms in the model, using pristine graphene as a reference system. The shape of the peak in Figure 5 was obtained by summing over each atomic contribution, with Gaussian broadening (with arbitrary constant width). The carbon atoms that have C−O bonds typically give rise to the peak on the positive side of the plots in (Figure 5). The other carbon atoms have varying shifts with positive and negative signs, leading to a broad peak on the right side of the C−O peak. For an oxygen concentration of 30%, the peak corresponding to the carbons that had no oxygen neighbors shifted to 0.66 eV, perhaps due to the loss of aromatic bonding.

The shifts of the C–O. peak relative to graphene ($\Delta BE^{C–O}$) are shown in Figure 1 as blue circles, in addition to the experimental difference between C–O and C=C ($sp^{2}$) energies. The increase in the shift follows a parabolic trend $\Delta BE^{C-O}=A{\left(c_{O}-c_{l}\right)}^{2}+\Delta_{0}$, where $c_{O}$ is the fractional oxygen concentration, A=52.3 eV, $c_{l}$ = 0.122, and $\Delta_{0}$ = 1.22 eV. For an oxygen concentration above 20%, the C1s core level shift relative to graphene increased until almost reaching 4 eV, which is in good agreement with the range of values attributed to single C−O bonds in GO experiments.

Additionally, Figure 1 shows the difference between the C1s core level shifts for the carbon atoms with C−O bonds, and those for the other carbon atoms ($\Delta BE^{C–O}-\Delta BE^{C–C}$). This is compared with the difference between the experimental XPS energies for the C–O and the C−C $sp^{2}$ peaks.

We considered the possibility that the C−O peak observed in XPS could also drift in the presence of hydroxyl groups. Hydroxyl groups are formed by protonation of epoxy groups of GO in water, and they may contribute to the C1s XPS ‘C−O’ peak. However, for the GO models in vacuum, it is only possible to add the first neutral hydroxyl, and additional hydroxyl groups are not bound. We have not considered charged species, as the Coulomb interactions between multiple charges and the core hole in a periodic system would introduce additional sources of error that are difficult to quantify. We have also considered the analogous shift of the carbonyl group, responsible for a minor C1s XPS peak in the samples. The displacement of the edge carbonyl C=O C1s core level shifts with the oxygen concentration is given in Supplementary Information S2.

In conclusion, we propose a technique for accurate measurement of the degree of oxidation of GO using XPS. The highest oxygen-related C1s XPS peak in graphene, the C−O peak, was attributed to the epoxy or hydroxyl. For GO with larger oxygen concentrations, the C−O C1s XPS peak increased and drifted towards higher energies with the oxygen concentration. We find that this drift can be well described by an approximate model of graphene covered by randomly distributed epoxy groups in varying concentrations. The shift energy can be obtained with remarkable accuracy from Equation (1), despite the fact that other functional groups were present in experiment and not in the theoretical model. We suggest that the energy position of the C−O C1s XPS peak can be used to measure the oxygen present at the basal plane of GO. This technique is also accurate for commercial samples of GO. Naturally, the better the quality of the sample, the closer the expected agreement to the theoretical model.

This technique does not replace the use of XPS wide survey spectra; however, it is complementary, and it offers advantages as a qualitative analytical method for fast determination, e.g., in an industrial context. The determination of the atomic fraction of oxygen from the survey spectra requires knowing the sensitivity factors or each element and type of photoelectron, representing the relative intensity of the peaks. However, such sensitivity factors depend on the energy-dependent transmission of the instrument for a given operating mode [18]. Further, the analysis requires the definition of the peaks and background subtraction, and it is complicated by the presence of plasmon losses and shake-up structures. In contrast, while the determination of the degree of oxidation from the C1s C−O peak shift still requires fitting of the C1s fine structure peaks, the energy of the peak position gives immediate information on how oxidized a sample is.

## 1. Methods

### 1.1. Materials Preparation

Graphene oxide dispersions were prepared by direct oxidation of graphene flakes using a modified Hummer’s method [6,8]. Graphene flakes were supplied by 2D Materials Pte Ltd., and the other reagents were purchased from Sigma-Aldrich. Graphene flakes were added to concentrated H${}_{2}$SO${}_{4}$, and the systems were cooled to 2 ${}^{\circ }$C, followed by slow addition of KMnO${}_{4}$. The reactions were further stirred at room temperature for different periods of time, followed by cooling back to 2 ${}^{\circ }$C. Finally, the systems were diluted in aqueous media, followed by the addition of H${}_{2}$O${}_{2}$ (35%). The resulting GO suspensions were washed with with 10% HCl, and dialysis was performed until pH 5.

### 1.2. XPS

Samples were drop-casted on silicon (Si) substrates for XPS analysis. The measurements were performed with Kratos AXIX Ultra (Kratos Analytical Ltd.) equipment with a mono-chromatic source, Al K*a* h*v* = 1486.81 eV. Calibration using a Shirley-type background, peak fitting, and quantification were carried out using Casa-XPS software (version 2.1.19). The percentage of oxygen was obtained from the oxygen peak intensity in the survey spectra [18].

### 1.3. First-Principles Calculations

First-principles calculations were based on the framework of DFT, as implemented in the Quantum Espresso package [19]. The PBE [20] exchange and correlation energy functional were used. Ultra-soft pseudo-potentials were used for carbon and oxygen [15], while a norm-conserving Troullier–Martins pseudo-potential was used for hydrogen [21]. We employed a plane wave basis set with kinetic energy cutoffs at 40 Ry to describe the electronic wave functions. The Brillouin zone was sampled using a Γ-centered 6 × 6 × 1 Monkhorst–Pack (MP) grid [22] for all calculations. A supercell periodicity of 20 Å in the direction perpendicular to the monolayer was used to avoid spurious interactions between replicas of the system.

## Figures and Tables

**Figure 1:**
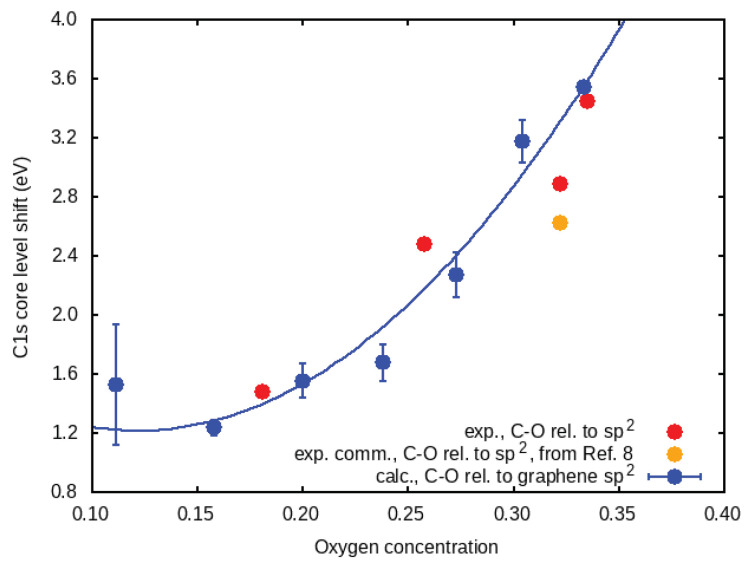
C1s core level shifts for carbon neighbors of epoxy oxygen (>O) vs. oxygen concentration. The theoretical values are for infinite graphene oxide with all oxygen in epoxy configuration, and they are referenced to graphene. The experimental oxygen concentration is obtained from the XPS survey spectrum. For comparison, we also show a commercial graphene sample. The calibration line is $\Delta E_{C1s}=A{\left(c_{O}-c_{l}\right)}^{2}+E_{0}$, where $c_{O}$ is the fractional oxygen concentration, A=52.3 eV, $c_{l}$ = 0.122, and $E_{0}$ = 1.22 eV.

**Figure 2:**
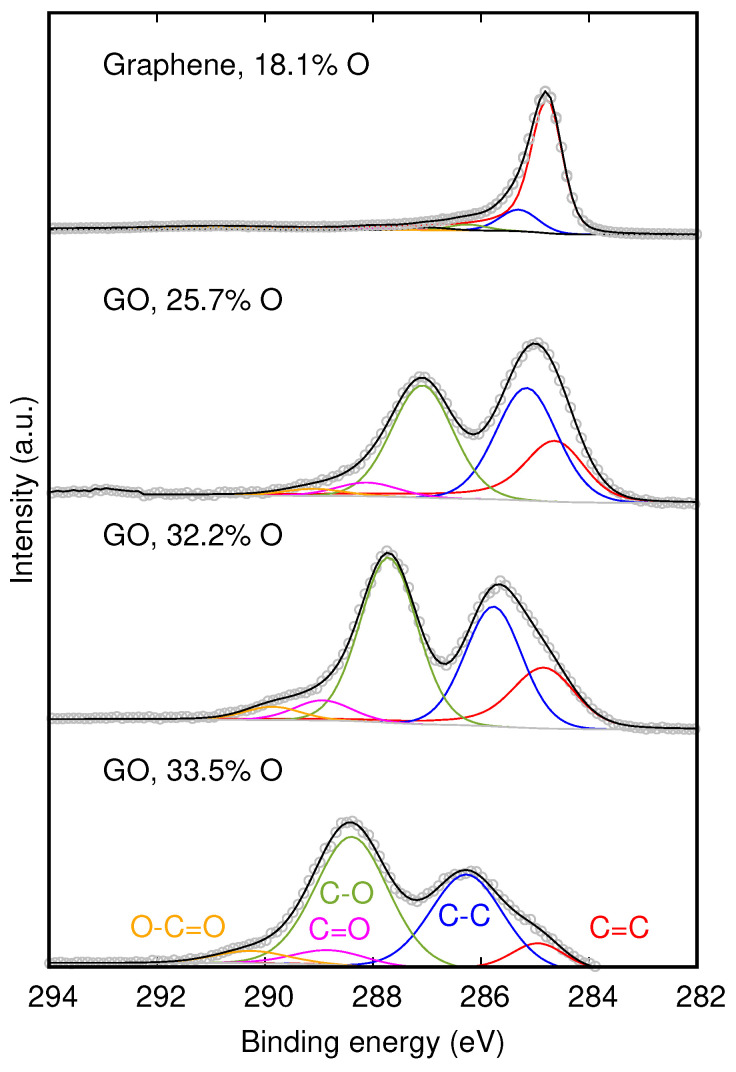
High-resolution C1s XPS spectra of graphene and graphene oxide with increasing oxygen concentration.

**Figure 3:**
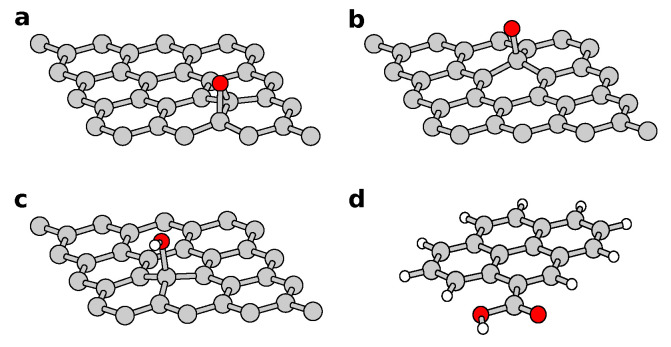
Epoxy (**a**), carbonyl (=O) (**b**), and hydroxyl (–OH) at the basal plane or (**c**) at the edge, and carboxyl (–COOH) (**d**) at the edge. Oxygen, carbon, and hydrogen are represented in red, gray, and white, respectively.

**Figure 4:**
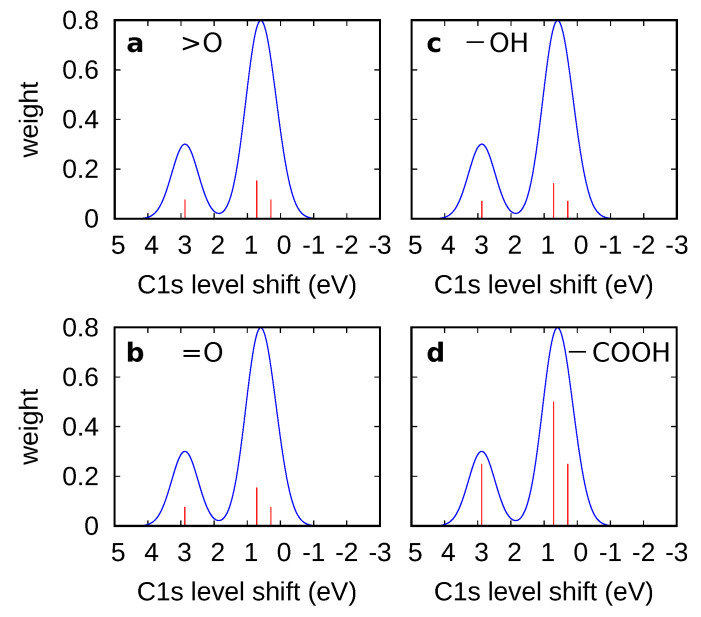
Calculated C1s core level shifts for oxygen defects in graphene: (**a**) epoxy (>O), (**b**) carbonyl (=O) on the basal plane, (**c**) hydroxyl on the basal plane (−OH), and (**d**) edge carboxyl (−COOH).

**Figure 5:**
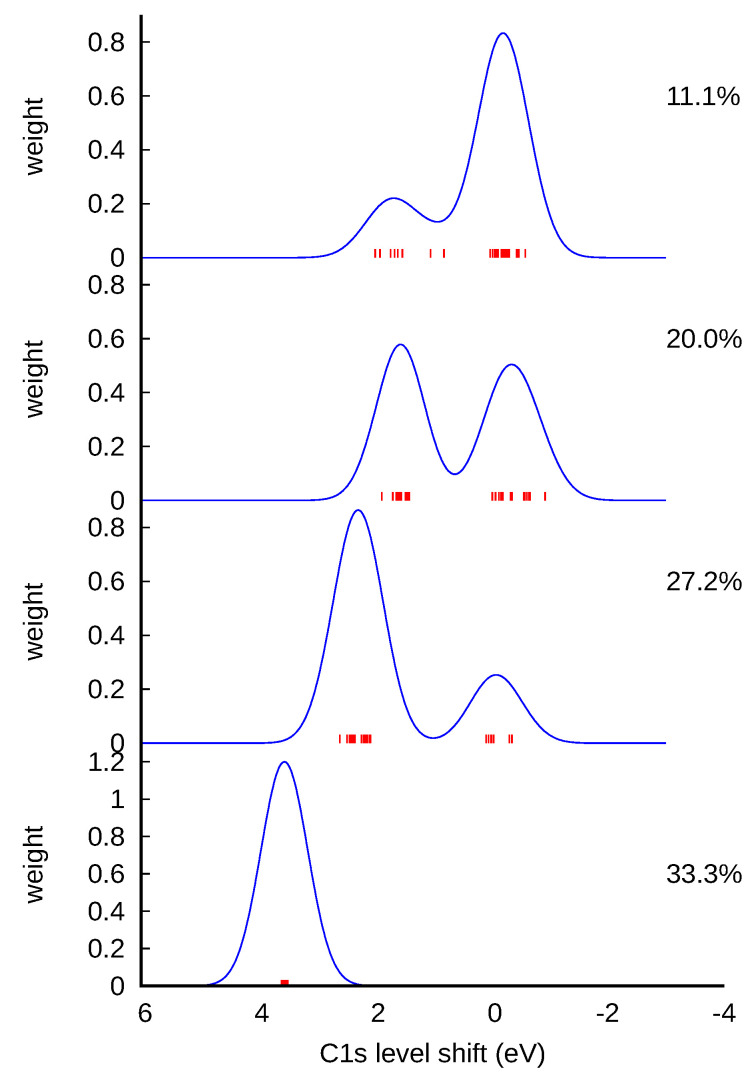
C1s core level shifts for oxygen defects in infinite graphene oxide, for different oxygen concentrations (given as percentage in the top right corner), with all oxygen in epoxy (>O) configurations.

## Data Availability

Data is contained within the article and supplementary material.

## Supplementary Materials

The following are available at https://www.mdpi.com/2079-4991/11/3/560/s1, Table S1: XPS data. Figure S1: Calculated XPS of edge carbonyl. Figure S2: Infra-Red spectra.
