# Supplementary material for: The Degree of Oxidation of Graphene Oxide
Source: Nanomaterials (Basel). 2021 Feb 24;11(3):560. doi: 10.3390/nano11030560 (PMC7995973; doi:10.3390/nano11030560)
Supplement: Supplementary file 1 [file nanomaterials-11-00560-s001.pdf]

The degree of oxidation of graphene oxide: Supplementary Information

A. Carvalho
Centre for Advanced 2D Materials, National University of Singapore, 117542 Singapore[*]

M. C. F. Costa
Centre for Advanced 2D Materials, National University of Singapore, 117542 Singapore and Materials Science and Engineering, National University of Singapore, 117575 Singapore

V. S. Marangoni
Centre for Advanced 2D Materials, National University of Singapore, 117542 Singapore

Ng Pei Rou
Centre for Advanced 2D Materials, National University of Singapore, 117542 Singapore

Nguyen Thi Le Hang
Centre for Advanced 2D Materials, National University of Singapore, 117542 Singapore

A. H. Castro Neto
Centre for Advanced 2D Materials, National University of Singapore, 117542 Singapore and Materials Science and Engineering, National University of Singapore, 117575 Singapore

[*]Electronic address: carvalho@nus.edu.sg

S1. XPS

TABLE S1: Binding energy (eV) of the deconvoluted C1s XPS peaks and their relative percentage area (in parentheses) for graphene and GO with different oxidation degree. Oxygen percentages were obtained from the Survey spectra.

Sample	[O](%)	C=C (sp^2)	C-C (sp^3)	C-O	C=O	O-C=O	π-π^*
Graphene	18.1	284.76 (81.8%)	285.30 (10.5%)	286.24 (2.8%)	286.99 (1.4%)	287.77 (0.7%)	290.90 (2.8%)
GO #1	25.7	284.60 (26.7%)	285.15 (33.6%)	287.01 (33.5%)	288.17 (4.0%)	289.10 (2.1%)	
GO #2	32.2	284.82 (22.1%)	285.77 (29.4%)	287.71 (40.4%)	288.93 (4.9%)	289.86 (3.2%)	
GO #3	33.5	284.94 (9.4%)	286.26 (34.3%)	288.39 (46.6%)	288.79 (5.3%)	290.26 (4.4%)	

S2. XPS of edge carbonyl in GO

The C1s core level shifts for the carbon nearest to an edge carbonyl were calculated using the edge model (Fig. 2). The oxygen content of the graphene oxide was modelled by parallel oriented epoxy oxygen, occupying randomly the sites represented as crossed red circles in the figure. Edge terminating atoms were not considered in the calculation of the oxygen concentration.

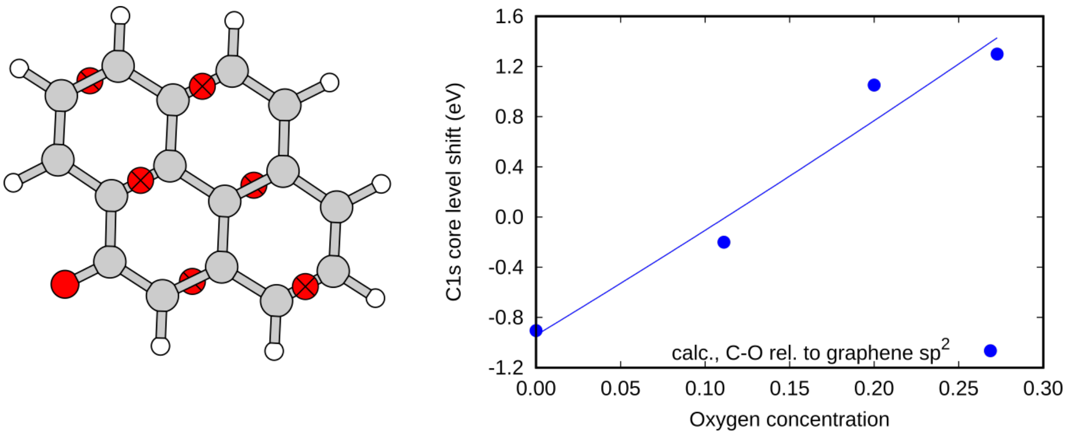

FIG. 1: C1s core level shifts vs. concentration for the edge carbonyl (=O), represented as a red circle on the left. Crossed red circles represent oxygen atoms added to increase the oxygen concentration.

S3. Fourier Transform Infra-Red (FTIR) spectra

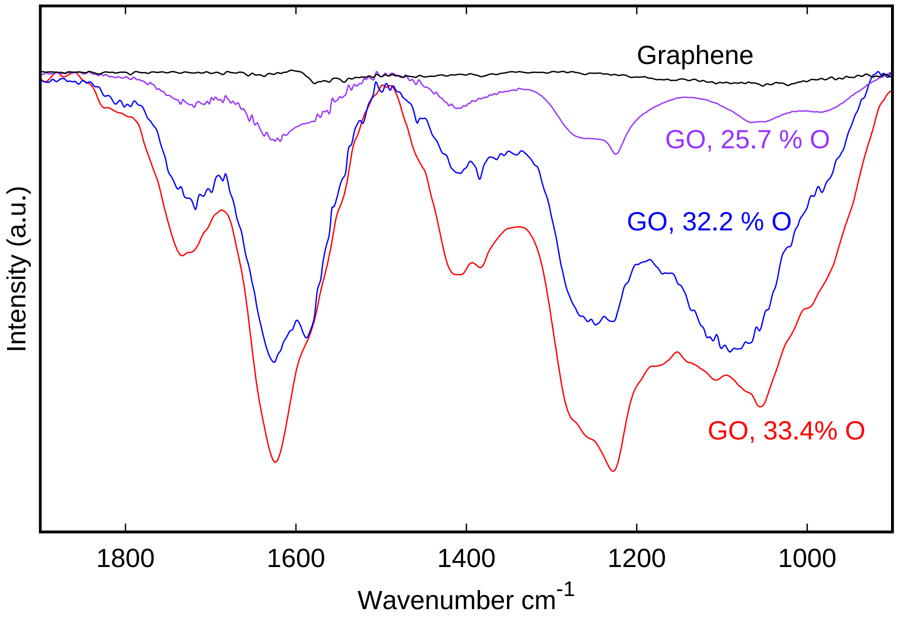

FIG. 2: FTIR spectra for the graphene and graphene oxide samples with different oxygen concentrations.

The FTIR spectra are typical for graphene oxide structures and are normalized with respect to the highest intensity band at 3400 cm^{-1} (which are hydrogen-related, and are not shown). The resonances at 1054, 1260 and 1418 cm^{-1} are attributed to the C-OH stretching, C-O stretching and O-H deformation vibrations, respectively. The 1260 cm^{-1} is the epoxy symmetric stretch.
